# Proteomic Analyses Reveal that Sky1 Modulates Apoptosis and Mitophagy in *Saccharomyces cerevisiae* Cells Exposed to Cisplatin

**DOI:** 10.3390/ijms150712573

**Published:** 2014-07-15

**Authors:** Silvia Rodríguez-Lombardero, M. Esther Rodríguez-Belmonte, M. Isabel González-Siso, Ángel Vizoso-Vázquez, Vanessa Valdiglesias, Blanca Laffón, M. Esperanza Cerdán

**Affiliations:** 1 EXPRELA Group, Department of Cellular and Molecular Biology, University of A Coruña, Campus A Coruña, A Coruña E15071, Spain; E-Mails: silviarlomb@gmail.com (S.R.-L.); belmonte@udc.es (M.E.R.-B.); migs@udc.es (M.I.G.-S.); avizoso@udc.es (A.V.-V.); 2 DICOMOSA Group, Department of Psychology, Area of Psychobiology, University of A Coruña, Campus A Coruña, A Coruña E15071, Spain; E-Mails: vvaldiglesias@udc.es (V.V.); blaffon@udc.es (B.L.)

**Keywords:** Bmh1, Bmh2, chemoresistance, yeast, cancer

## Abstract

Sky1 is the only member of the SR (Serine–Arginine) protein kinase family in *Saccharomyces cerevisiae.* When yeast cells are treated with the anti-cancer drug cisplatin, Sky1 kinase activity is necessary to produce the cytotoxic effect. In this study, proteome changes in response to this drug and/or *SKY1* deletion have been evaluated in order to understand the role of Sky1 in the response of yeast cells to cisplatin. Results reveal differential expression of proteins previously related to the oxidative stress response, DNA damage, apoptosis and mitophagy. With these precedents, the role of Sky1 in apoptosis, necrosis and mitophagy has been evaluated by flow-cytometry, fluorescence microscopy, biosensors and fluorescence techniques. After cisplatin treatment, an apoptotic-like process diminishes in the ∆*sky1* strain in comparison to the wild-type. The treatment does not affect mitophagy in the wild-type strain, while an increase is observed in the ∆*sky1* strain. The increased resistance to cisplatin observed in the ∆*sky1* strain may be attributable to a decrease of apoptosis and an increase of mitophagy.

## 1. Introduction

Diverse derivatives of platinum are used as therapeutic agents in cancer treatment [1,2,3]. Among them, cisplatin, *cis*-diaminodichloroplatinum, a neutral square planar complex of platinum that forms adducts with DNA. In mammals, the intra-strand cross-link is the major lesion caused by cisplatin-induced DNA damage and it is primarily repaired via the nucleotide excision repair (NER) system. In addition to NER, cisplatin can also induce transcription-coupled repair (TCR), since the intra-strand crosslinks stall RNA polymerase II. The homology-directed DNA repair (HDR), which allows error-free repair of the double-strand breaks after the excision of cisplatin–DNA adducts, is also implied. On the contrary, the mismatch repair (MMR) responds to cisplatin-induced DNA damage increasing cytotoxicity. Cisplatin also exhibits a high affinity towards sulfur donors, such as cysteine and methionine, forming stable Pt–S bonds, which compete with the DNA binding, thus decreasing the cytotoxicity of cisplatin (reviewed in [4]). The toxicity of cisplatin also affects yeast survival and therefore *Saccharomyces cerevisiae* has been used as a convenient eukaryotic model to find genes related to cisplatin resistance [5] or sensitivity [6]. However, molecular mechanisms of cisplatin action in yeast have been scarcely studied. Cunha *et al*. have recently reported that cisplatin induces an active form of cell death in yeast and increases DNA condensation and fragmentation, but it does not cause significant loss in the mitochondrial membrane potential [7].

Eukaryotic SRPKs are constitutively active serine-arginine kinases, which display remarkable substrate specificity compared to other kinases, and they have been related to RNA processing. Sky1 is the only member of the SRPK family in *S. cerevisiae* and its enzymatic activity is required for the cytotoxicity produced by cisplatin [8]. Among the known protein substrates of Sky1 are Npl3 [9,10] and Gbp2 [11]. It has been reported that Hrb1 is also phosphorylated by Sky1 *in vitro* [12]. These three substrates are identified as shuttling RNA binding proteins, RNA binding proteins that remain associated to mRNA during export from the nucleus. Alignment of the phosphorylation targets in the two known substrates of Sky1, Gbp2 and Npl3 suggests a Sky1-specific recognition site fitting the consensus R(E/S)RSP(T/V)R [13], which is slightly different to the mammalian SRPK recognition site RSRSRSR [14]. The function of the *SKY1* gene in the modulation of cisplatin sensitivity is also conserved in *SRPK1* and *SRPK2*, their human homologs. Inactivation of *SRPK1*, using an antisense approach, induces cisplatin resistance in a human ovarian carcinoma cell line [8]. Besides, heterologous expression of *SRPK1* in a ∆*sky1* yeast strain is able to complement its phenotype of resistance to cisplatin [8]. Phosphorylation mediated by SRPK1 and SRPK2 kinases signals caspase-8 pre-mRNA splicing in response to cisplatin and it also determines whether cells undergo apoptosis or G(2)/M cell cycle arrest [15].

In yeast, deletion of the *SKY1* gene confers resistance to cisplatin but it does not produce changes in intracellular levels of this compound or changes in DNA platination [16]. Although the *∆sky1* cells have a hypermutator phenotype, thus suggesting a role of Sky1 in specific DNA repair pathways [16], the mechanisms by which it modulates cisplatin sensitivity are unknown. Sky1 is also related to polyamine import into the cell and to the maintenance of the membrane potential, although these processes have not been related to cisplatin sensitivity [17,18]. In this work we studied, by a proteomic approach, the response of *S. cerevisiae* to cisplatin treatment and/or *SKY1* deletion. Among the proteins identified in this analysis Bmh1/2 and Mmi1 (Tma19) have been previously related to apoptosis and mitophagy. To test the influence of Sky1 function on these processes, necrotic-apoptotic events and mitophagy were also measured. Results confirm that Sky1 is related to both apoptosis and mitophagy in response to cisplatin exposure of yeast cells.

## 2. Results and Discussion

2.1. 2-D Difference Gel Electrophoresis (DIGE) Analysis

Cells obtained from three independent cultures of the *S. cerevisiae* strains W303 and its isogenic *Δsky1* derivative, untreated or after cisplatin treatment, were used for protein isolation, phospho-protein enrichment and 2-D difference gel electrophoresis (DIGE) analyses. In the design of the proteomic approach we carried out a selection of proteins previous to 2-D DIGE analyses, by using the Diamond Phosphoprotein Enrichment Kit (Invitrogen, Carlsbad, CA, USA) as described in the Experimental Section. The use of this kit allows the enrichment in acidic proteins, these including phosphoproteins. The rationale of this strategy was based in the knowledge that Npl3 and Gbp2, the two characterized substrates of Sky1 *in vivo*, are acidic proteins. Npl3 has a theoretical *pI*, calculated from its amino acid content, of 5.35 and the experimentally determined value decreases to 4.58 [19]. Gbp2 has a theoretical *pI* of 6.16.

**Figure 1:**
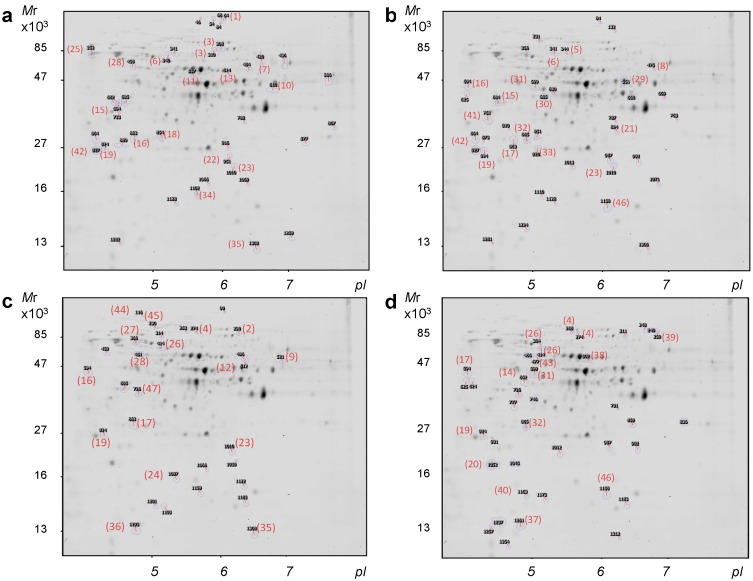
Representative phosphoproteome map showing the proteins with significant changes (**a**) in the *S. cerevisiae* W303-1A haploid strain after treatment with cisplatin; (**b**) in the W303-1A-derived *Δsky1* mutant after treatment with cisplatin; (**c**) in the W303-1A strain *versus* its *Δsky1* mutant; (**d**) in the W303-1A strain *versus* its *Δsky1* mutant, after treatment with cisplatin. Proteins were resolved in the 3–11 (nonlinear) pH range in the first dimension and on 12% acrylamide gels in the second dimension. Proteins identified by MALDI (Matrix-assisted laser desorption/ionization)-TOF (Time of Flight)/TOF mass spectrometry are listed in Table 1, where ID numbers correspond to the numbered spots in this figure (red numbers in brackets).

Images were obtained from each gel, corresponding to Cy2-, Cy3- and Cy5-labeled samples. In order to statistically analyze the protein changes, 12 gel images (Cy3 and Cy5) were analyzed using Progenesis SameSpots V3.2 software (Nonlinear Dynamics, Newcastle upon Tyne, UK) available through [20]. Intergel normalization was also carried out using Progenesis SameSpots V3.2 software (Nonlinear Dynamics, Newcastle upon Tyne, UK). In our study inter-image analysis allowed matching of 953 spots, which represents a reasonable number of detected proteins in comparison to previous data about the yeast phosphoproteome size, which varies from 160 to 1513 in different reports [21,22,23]. Proteins with statistically significant changes in these analyses are shown in Figure 1. When proteins from the W303 strain with and without cisplatin treatment were compared, 16 spots increased after treatment and 24 decreased according to this analysis (Figure 1A). When proteins from the *Δsky1* strain with and without cisplatin treatment were compared, 25 spots were increased and 14 decreased after treatment (Figure 1B). Comparison of proteins from the W303 and *Δsky1* strains without treatment showed that 12 spots increased in the mutant while 18 decreased (Figure 1C). Finally, comparison of proteins from the W303 and *Δsky1* strains with cisplatin treatment showed that 22 spots increased in the mutant while 14 decreased (Figure 1D).

### 2.2. Identification of the Differentially Quantified Proteins

To identify the proteins showing statistically significant changes in the previous comparisons, selected spots were digested in-gel, and analyzed by MALDI (Matrix-assisted laser desorption/ionization)-TOF (Time of Flight)/TOF. We considered statistically significant only those changes with ANOVA *p* value statistically significant. The searches for peptide mass fingerprints and tandem MS spectra were performed in the UniProt knowledgebase, by searching in the UniProtKB/Swiss-Prot [24] database. In spot 610 (see Table 1), two different proteins were identified. Table 1 shows detailed information comprising experimental and theoretical molecular weight and *pI* values, accession numbers and identification parameters of the proteins that are differentially quantified under the tested comparisons. The observed differences may be caused by differential protein expression or by differential extraction caused by *pI* changes attributable to posttranslational modifications. Comparison between fold changes observed in Table 1 and those observed in transcriptome studies (Table S1) reveals that variation in protein levels are positively correlated with mRNA levels only in few cases (*SHM2*, *MET6*, *ADO1*, *ADH1* or *ARO4*). For the rest, changes in protein levels are attributable to other regulatory mechanisms.

**Table 1:** Proteins with significant abundance changes in the proteome analyses.

Untreated Mutant Δ*sky1* *versus* Untreated Wild-Type								
Spot	Anova (*p*)	Fold	ID	Identification	Experimental		Predicted	
					*Mr*	*pI*	*Mr*	*pI*
924	0.05	−1.6	19	*YLR179C*	27	3.98	22.15	4.77
290	0.05	−1.3	2	*ACS2*; *YLR153C*	76	6.10	75.49	6.21
521	0.03	−1.3	9	*SHM2*; *YLR058C*	53	7.24	52.21	6.98
1010	0.05	−1.3	23	*GUK1*; *YDR454C*	22	5.98	20.64	6.63
1293	0.05	−1.3	36	*PFY1*; *YOR122C*	13	4.85	13.68	5.47
**Wild-Type Treated *versus* Untreated**								
**Spot**	**Anova (*p*)**	**Fold**	**ID**	**Identification**	**Experimental**		**Predicted**	
					***Mr***	***pI***	***Mr***	***pI***
310	0.02	1.3	3	*MET6*; *YER091C*	73	5.70	85.86	6.07
537	0.04	1.3	11	*LYS20*; *YDL182W*	51	5.58	47.10	6.84
684	0.02	−1.4	15	*ADO1*; *YJR105W*	41	4.36	36.37	4.99
610	0.05	−1.3	10	*ADH1*; *YOL086C*	45	6.89	36.85 40.01	6.21 6.47
**Mutant Δ*sky1* Treated *versus* Untreated**								
**Spot**	**Anova (*p*)**	**Fold**	**ID**	**Identification**	**Experimental**		**Predicted**	
					***Mr***	***pI***	***Mr***	***pI***
475	0.05	1.8	8	*UGP1*; *YKL035W*	54	6.89	55.99	6.98
824	0.03	1.7	21	*PRB1*; *YEL060C*	32	6.04	69.62	5.94
341	0.04	1.3	6	*STI1*; *YOR027W*	69	5.4	66.26	5.45
761	0.02	1.3	41	*BMH1/BMH2*	36	4.09	30.09	4.82
927	0.05	−1.5	42	*TMA19*; *YKL056C*	17	3.78	18.741	4.41
1010	0.01	−1.3	23	*GUK1*; *YDR454C*	22	5.98	20.64	6.63
684	0.05	−1.3	15	*ADO1*; *YJR105W*	41	4.36	36.37	4.99
**Treated Mutant Δ*sky1 versus* Treated Wild-Type**								
**Spot**	**Anova (*p*)**	**Fold**	**ID**	**Identification**	**Experimental**		**Predicted**	
					***Mr***	***pI***	***Mr***	***pI***
865	0.03	1.4	32	*HSP26*; *YBR072W*	30	5.11	23.88	5.31
1052	0.05	1.3	20	*AHP1*; *YLR109W*	19	4.21	19.11	5.01
924	0.01	−1.6	19	*YLR179C*	27	3.98	22.15	4.77

Spot, master protein spot number according to Figure 1A–D; ID, identification protein number. ID correspond to the red numbers (between brackets) in Figure 1; Fold = Average volume ratio, positive is fold increment and negative is fold decrement; Experimental *Mr* (kDa) and *pI* calculated by analysis of the gel images as explained in the text. Predicted *Mr* and *pI* according to protein sequence and Mascot search results.

### 2.3. Validation of the Experimental Design

To validate the adequacy of the methods used, the characteristics of the identified proteins were analyzed. Evaluation of the abundance of the identified proteins in our study (according to Ghaemmaghami *et al*. [25]) show that proteins ranging from 1600 to 264,000 molecules per cell are represented (Table S2). This result indicates that the method used for acidic/phosphorylated protein enrichment does not exclude capture of high or low expressed proteins, although the latter are less represented. As a shortcoming of the methods, comparative evaluation of the stability of the proteins detected in our study using the ProtParam tool from Expasy [26] shows that more stable proteins (instability index lower than 40) predominate over the unstable ones (Table S1). This may justify the absence of Npl3 and Gbp2, known substrates of Sky1 [9,10,11], among the detected proteins, since they have high instability indexes of 66.14 and 44.70 respectively. As expected, all the proteins identified in this study (with the exception of profilin and the uncharacterized protein encoded by YLR179C) are previously recognized phosphoproteins identified by conventional or phosphoproteomic analyses (data from PhosphoGrid database). However, among the identified proteins in our analyses we do not find a similar sequence to the one predicted for the Sky1 substrates, R(E/S)RSP(T/V)R; although caution about the robustness of this consensus is needed, because only two sequences were aligned for its definition [13]. Alternatively, other kinases may be regulated by Sky1 function and/or cisplatin treatment. The predicted and gel-based *Mr* and *pI* were also compared taking the spots from the four comparisons together (Figure S1). The *pI*s correlated poorly (*R* = 0.7118) suggesting a high degree of posttranslational modifications. This was expected, since acidic/phosphoprotein enrichment was done before DIGE analysis. Indeed, proteins are shifted towards the acidic region of the gels with most of them having an experimental *pI* less than 6.3.

### 2.4. Proteome Changes Produced by Δsky1 Deletion

The effect of the *Δsky1* deletion in absence of cisplatin treatment, in the conditions of our study, is depicted in Table 1. The previously characterized substrates of the Sky1 kinase are RNA binding proteins [10,11], however the proteins affected by Sky1 depletion in our study are related to other functions. The proteins that show lower levels in the mutant than in the wild-type are functionally related to DNA synthesis, chromatin modification and/or oxidative stress. Shm2 is involved in generating precursors for purine and pyrimidine biosynthesis [27]. Guk1, guanilate kinase, participates in the nucleotide metabolism [28]. Acs2 catalyzes histone acetylation [29]. The profilin Pfy1, which activates actin polymerization, increases the viability of cells exposed to oxidative stress [30]. Therefore, the proteomic analysis potentially extends the functions already assigned to Sky1 in previous studies.

### 2.5. Proteome Changes Produced by Cisplatin Treatment and Their Dependence on Sky1 Function

A comparison of data reported in Table 1 allows us to recognize those proteins that are regulated by the signal elicited by cisplatin treatment with reference to the expression of the *SKY1* gene. About the proteins whose expression change after cisplatin treatment (Met6, Lys20, Adh1 and Aro4), all are Sky1 dependent (a variation is observed after treatment in the wild-type but not in the ∆*sky1* mutant) with the exception of Ado1. This protein participates in nucleotide metabolism and it has been previously reported that stimulation of *de novo* synthesis of purine nucleotides increases cisplatin resistance [31].

The protein Met6, 5-methyltetrahydropteroyltriglutamate-homocysteine methyltransferase, catalyzes the transfer of a methyl group from methyltetrahydrofolate to homocysteine resulting in methionine formation. Lys20, homocitrate synthase, catalyzes the biosynthesis of l-lysine via aminoadipic acid. In *S. cerevisiae* Met6 and Lys20 have been previously related to oxidative stress. Methionine decay in *met6* mutants up-regulate protection to increased oxidative metabolism [32] and, oppositely, we may speculate that Met6-increase could cause decreased protection against oxidative stress. *LYS20* gene expression is also induced after H_2_O_2_ treatment [33]. Besides, the proteins Adh1, alcohol dehydrogenase 1, and Aro4, phosphor-2-dehydro-3-deoxyheptonate aldolase, have been previously related to the oxidative stress response [34,35]. In mammals it has been described that cisplatin treatment causes oxidative stress, which may even become the cause of cell-death [36,37].

### 2.6. Proteins Related to the Increased Resistance of Cisplatin in the Δsky1 Mutant

**Figure 2:**
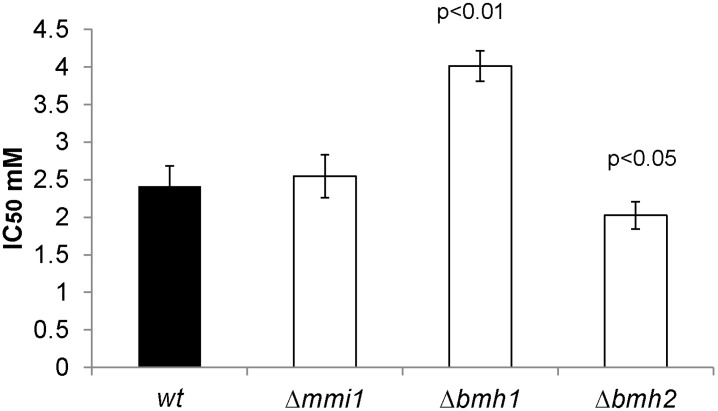
Resistance to cisplatin of *Δmmi1*, *Δbmh1*, *Δbmh2* and their parental wild-type strain (wt).

Although a role of Sky1 in specific DNA repair pathways has been suggested [16], the mechanisms by which the *Δsky1* mutant becomes more resistant to cisplatin, are unknown. A comparison of data reported in Table 1 allows us to recognize proteins that may contribute to this effect. Those that change their levels in the comparison of *Δsky1* cisplatin treated cells *versus* wild-type treated cells as well as those differenced in the comparison *Δsky1* treated*versus* untreated cells, but which do not change in the comparison wild-type treated *versus* untreated may be considered. Analysis of this group reveals six up-regulated proteins (Ugp1, Prb1, Sti1, Bmh1/2, Hsp26 and Aph1) and one down regulated (Mmi1, alias Tma19). Mmi1 has sequence homology to the human translationally controlled tumour protein (TCTP), which was originally found in tumor cells. Interestingly, TCTP is a critical survival factor that protects cancer cells from oxidative stress-induced cell-death [38]. Moreover, yeast Mmi1 has been related to resistance to oxidative stress and the deletion mutant displays resistance to hydrogen peroxide [39]. Mmi1 fused to GFP localizes to the cytoplasm and relocates to the mitochondrial outer surface upon oxidative stress [39]. Several among the up-regulated proteins have been also previously related to oxidative stress. This is the case of Aph1 [40], Ugp1, [32,34] and Hsp26 [34]. Hsp26 has also been related to DNA damage [41] and Bmh1 to the modification of histones [42]. Bmh1 levels also increase during DNA replication stress [43]. In Table 1 the unambiguous identification of spot 761 to determine whether Bmh1 or Bmh2 increased their levels in the *∆sky1* treated *versus* untreated cells was not possible by MS; this is due to the fact that these proteins differ only in a short stretch of amino acids at the carboxylic end, which unfortunately was not detected in the analysis of peptides. We tested if Bmh1 or Bmh2 are related to cisplatin toxicity in yeast. The null *∆bmh1* and *∆bmh2* strains were compared to an isogenic wild-type strain in their resistance to cisplatin. Results obtained reveal that the *∆bmh1* strain has increased resistance against cisplatin, while deletion of *BMH2* causes the contrary effect (Figure 2). These results confirm the functional significance of the change observed in the proteome data. They also indirectly indicate that the observed increase in cisplatin resistance observed in the *∆sky1* mutant might be attributed to Bmh2 up-regulation, considering that *∆bmh2* deletion has the opposite effect diminishing cisplatin resistance. In a similar analysis, *MMI1* deletion does not cause significant change in cisplatin resistance (Figure 2).

### 2.7. Cisplatin Increases Apoptotic-Like Events in Yeast Cells and Sky1 Modulates this Response

As discussed above, among the proteins which change their levels after cisplatin treatment of yeast cells, there are proteins previously related to the oxidative stress response and it is known that redox imbalance and mitochondrial dysfunction targets apoptosis and cell death [39]. Several proteins that are differentially expressed in *∆sky1* treated *versus* untreated cells (Table 1) have been previously related to apoptosis in yeast, like, Bmh2 and Mmi1. Bmh2, shows strong similarity to the ubiquitous and highly conserved 14-3-3 proteins which likely play a role in signal transduction [44] and protect against stress-induced apoptosis [45]. Mmi1 has been related to apoptosis induced by oxidative stress [39].

**Figure 3:**
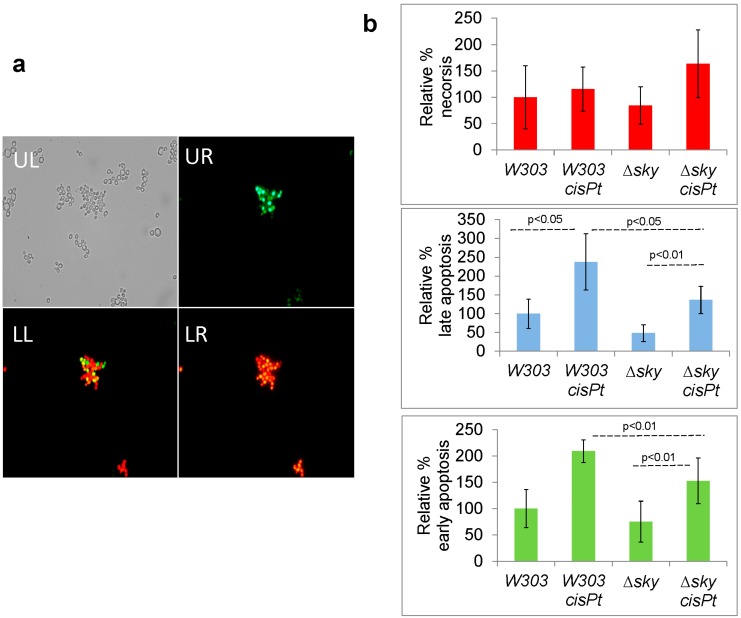
Apoptosis and necrosis induced by cisplatin (*cisPt*) in W303-1A (*W303*) and *∆sky1* strains (**a**) apoptosis or/and necrosis visualized by fluorescence microscopy (objective 40×): UL, Bright field; UR Green channel for YO-PRO^®^-1 staining; LR, Red channel for PI staining; LL Merge; (**b**) Quantification of necrosis, late apoptosis and early apoptosis by flow cytometry.

Cisplatin, like many other chemotherapeutic drugs, can induce apoptosis in mammals [4]. In yeast a programmed cell-death related to cisplatin treatment has been recently reported [7]. The changes, observed in the proteome analysis after cisplatin treatment and Sky1 depletion, suggest that Sky1 may modulate this cellular response. Figure 3A shows W303-1A yeast cells treated with cisplatin and stained with YO-PRO^®^-1 (Invitrogen, Carlsbad, CA, USA) and propidium iodide (PI). Figure 3B shows the quantification of data by flow cytometry. We have verified that cisplatin treatment increments YO-PRO^®^-1 staining, which reports apoptotic events. Sky1 depletion decreases the number of early and late apoptotic cells without affecting necrosis.

### 2.8. Deletion of SKY1 Increases Mitophagy in Yeast Cells after Cisplatin Treatment

The activation of the retrograde response in yeast causes a wide variety of changes in the expression of nuclear genes encoding mitochondrial, cytoplasmatic or peroxisomal proteins, which reorganize the cellular metabolism and compensate for mitochondrial dysfunction [46]. The retrograde response may be initially a strategy for extending life span, although in the long term it can ultimately contribute to reduce genome stability and produces cell death [47]. The retrograde response may be activated together with mitophagy and Bmh1 has been previously related to the regulation of this process [46].

**Figure 4:**
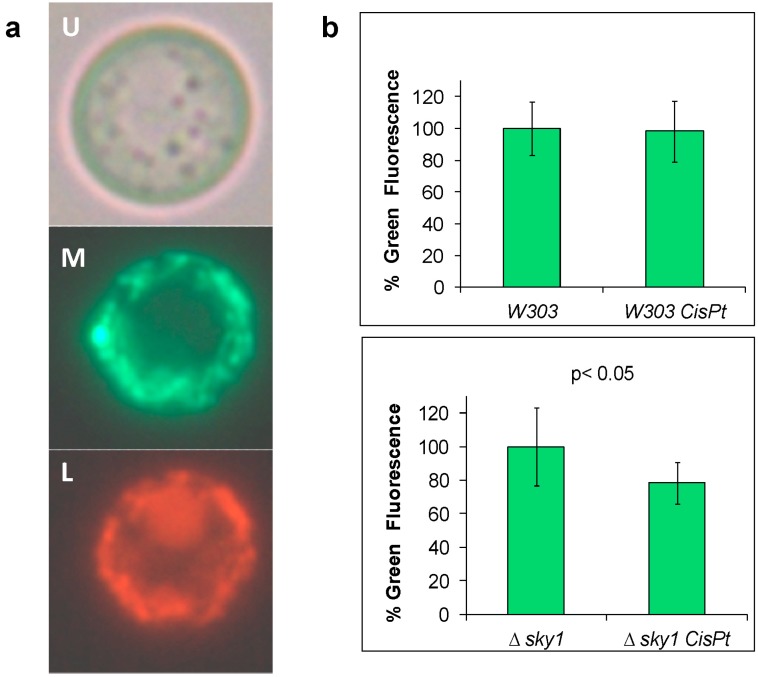
Mitophagy in W303-1A (*W303*) and *∆sky1* strains after cisplatin (*cisPt*) treatment. (**a**) Visualization (objective 100×) of mitophagy in W303 cells treated with 1200 µM cisplatin; U, Bright field; M, Green channel for phluorin; L, Red channel for the Ds. Red T3 protein; (**b**) Quantification of the green fluorescence.

We studied if mitophagy is implicated in the response to cisplatin in yeast and its dependence on Sky1 function. Mitophagy was optically visualized by the method previously described [48] and we observed the presence of mitochondria into vacuole in all the tested strains, which indicates the correct performance of the fluorescent biosensor; one example is shown in Figure 4A. A six fold increase in the percentage of cells showing mitophagy was recorded by direct count in the microscope when comparing the mutant strain treated with cispaltin *versus* untreated. No significant change was observed in the same conditions for the W303-1A strain. Quantification of green fluorescence by the method described in the Experimental Section reveals that mitophagy is not increased in reference to the untreated cells in the wild-type strain (Figure 4B). Sky1 depletion decreases the green fluorescence in cisplatin treated cells compared to the untreated cells (Figure 4B), therefore confirming increased mitophagy, which argues in favor of the participation of Sky1 in this response. The increased resistance to cisplatin observed in the *∆sky1* strain may be partially attributable to an increase of mitophagy.

## 3. Experimental Section

### 3.1. Strains and Culture Conditions

*S. cerevisiae* haploid W303-1A (MATa *ade2-1 can1-100 leu2-3*, *112 trp1-100 ura3-52*) and the isogenic *Δsky1* mutant strains were used in this work. The construction of the *Δsky1* strain has been previously described [49]. The strains *Δmmi1*, *Δbmh1*, *Δbmh2*, and their isogenic parental strain BY4741 were obtained from EUROSCARF [50]. Biological replicates of cultures and treatments were run in triplicate. The two yeast strains (ten independent colonies from each strain obtained from the −80 °C stocks) were pre-cultured overnight in 20 mL of synthetic medium (SD) prepared as previously described [51]. The following day the cells were inoculated, at an initial OD_600_ of 0.05, in 500 mL SD and grown during 14 h at 30 °C and with agitation at 250 rpm. Then, the cultures from each strain were split in two of 250 mL (control and cisplatin treatment) and cisplatin was added to the treated cultures at a final concentration of 600 µM. The treatment was done at 30 °C and with agitation at 250 rpm over four hours in darkness. The concentration of cisplatin and the time course of the treatment were previously established in trial experiments with the selected yeast strains. In the conditions used in these experiments cell survival in the treated cells was over 80% and an increase of 10%–15% survival was observed in the W303–*Δsky1* mutant in reference to the W303-1A strain.

### 3.2. Protein Extracts Preparation and Phosphoprotein Enrichment

Cells were collected by centrifugation at 7000 rpm in a microcentrifuge at 4 °C over 10 min and re-suspended in 1 mL/g (wet-weight) of lysate-buffer supplemented with 4 µL/mL protease inhibitors, both provided in the Diamond Phosphoprotein Enrichment Kit (Invitrogen, Carlsbad, CA, USA). Mechanical lysis was performed using glass beads (to 1/2 volume, acid-washed, 45 µM from Sigma–Aldrich, St. Louis, MO, USA) and hard vortex agitation in 8 pulses of 20 s each followed by 40 s in ice. After centrifugation at 8000 rpm in a microcentrifuge at 4 °C for 15 min, supernatants were saved. Eight microliter of endonuclease (included in the kit) was added and samples were incubated for 30 min on ice, with repeated middle vortex agitation after 5 min periods. Following centrifugation at 10,000× *g* at 4 °C for 20 min, the pellets were removed. Phosphoprotein enrichment and elution were performed with the specific columns and buffers of Diamond Phosphoprotein Enrichment Kit (Invitrogen, Carlsbad, CA, USA) and following the supplier indications. The proteins were precipitated with trichloroacetic acid (TCA) and centrifuged at 9000× *g*. The pellet was solubilized by 1 h incubation with gentle agitation in an isoelectric focusing compatible urea lysis buffer [52]. To quantify protein, triplicate 2–4 µL samples of each extract were diluted to 50 µL with water and the Pierce BCA (bicinchoninic acid) technique was used. Correct quantification was confirmed by loading 5 µg of each sample on a standard SDS-PAGE gel and subsequent silver-staining (Figure S2A). One of the samples was also loaded on a DIGE gel and silver-stained (Figure S2B).

### 3.3. DIGE Experimental Design and Protein Labeling

The proteomic comparison of the four experimental conditions was performed across six DIGE gels using 45 µg of total protein fraction per CyDye™ gel and three biological replicates for each condition. The 12 individual samples were generated from four experimental conditions (W303-1A not treated, W303-1A treated, *∆sky1* treated and *∆sky1* treated). Proteins in each sample were fluorescently tagged with a set of matched fluorescent dyes according to the manufacturer’s protocol for minimal labeling (Figure S2C). To ensure that there were no dye-specific labeling artifacts, sample replicates in different gels were labeled with either Cy3 or Cy5, whereas the pooled standard sample was labeled with Cy2. The standard pool was prepared by pooling 45 µg of proteins from each sample prior to labeling. In every case, 360 pmol of dye was used for 45 µg of proteins. Labeling was performed for 30 min on ice in darkness, and the reaction was quenched with 1 µL of 10 mM l-lysine for 10 min under the same conditions.

### 3.4. 2-DE and Imaging of Cy-Labeled Proteins

The six pairs of Cy3- and Cy5-labeled samples (each containing 45 µg of protein) were combined and mixed with a 45 µg aliquot of the Cy2-labeled standard pool. The mixtures containing 135 µg of protein were diluted 1:1 with rehydration buffer (7 M urea, 2 M thiourea, 4% CHAPS, 4% ampholytes pH 3–11, and 2.4% DeStreak reagent from GE-Healthcare Life Sciences, (Branford, CT, USA). The IPG (immobilized pH gradient) strips (24 cm, pH 3–11 non-linear) were rehydrated overnight with 450 µL of a rehydration buffer as above but with 2% ampholytes, 0.002% bromophenol blue, and 97 mM DeStreak reagent. The labeled samples were then applied to the strips by cup-loading on an IPGphor II IEF system (GE Healthcare). Isoelectric focusing was carried out using the following conditions: 1 h at 120 V, 1 h at 500 V, 1 h at 1000 V, gradient to 4000 V in 1 h, and finally 12 h at 4000 V. Prior to the second dimension run, the strips were equilibrated first for 15 min in equilibration buffer (100 mM Tris-HCl (pH 8.0), 6 M urea, 30% glycerol, and 2% SDS) with 2% DTT and then for 15 min in the same buffer supplemented with 2.5% iodoacetamide and 0.002% bromophenol blue. The equilibrated strips were transferred onto 12% homogenous polyacrylamide gels casted in low fluorescence glass plates using an Ettan-DALT six system (GE Healthcare, Wilmington, MA, USA). Electrophoresis was run at 2 watts/gel and 20 °C for about 17 h. The differentially labeled co-resolved proteins within each gel were imaged at 100 dots/inch resolution using a DIGE Imager scanner (GE Healthcare). Cy2-, Cy3-, and Cy5-labeled images of each gel were acquired at excitation/emission values of 488/520, 523/580, and 633/670 nm, respectively. Gels were scanned directly between the glass plates, and the 16-bit image file format images were exported for data analysis. After imaging for Cy-Dyes, the gels were removed from the plates and stained with colloidal Coomassie, following standard procedures.

### 3.5. Image Acquisition and DIGE Data Analysis

Semi-automated image analysis was performed with Progenesis SameSpots V3.2 software (Nonlinear Dynamics, Newcastle upon Tyne, UK). Image quality control was first performed to identify saturated spots. Multiplexed analysis was selected for DIGE experiments and a representative gel image was chosen as reference. Spots were detected and their normalized volumes were ranked on the basis of ANOVA *p*-values and fold changes. In the proteomic analyses, normalization tools and statistical package from SameSpots and ProteinPilot software (AB Sciex, Framingham, MA, USA) were employed.

### 3.6. MS Analysis of the Gel Spots

The gel spots of interest were manually excised and transferred to microcentrifuge tubes. Samples selected for analysis were in-gel reduced, alkylated and digested with trypsin according to the method of Sechi and Chait [53]. The samples were analyzed using the MALDI (Matrix-assisted laser desorption/ionization)-TOF (Time of Flight)/TOF mass spectrometer 4800 Proteomics Analyzer (AB Sciex, Framingham, MA, USA) and 4000 Series Explorer™ Software (AB Sciex). Data Explorer version 4.2 (AB Sciex) was used for spectra analyses and for generating peak-picking lists. All mass spectra were internally calibrated using autoproteolytic trypsin fragments and externally calibrated using a standard peptide mixture (Sigma–Aldrich). TOF/TOF fragmentation spectra were acquired by selecting the 10 most abundant ions of each MALDI-TOF peptide mass map (excluding trypsin autolytic peptides and other known background ions).

### 3.7. Database Search

The mono-isotopic peptide mass fingerprinting data obtained by MS and the amino acid sequence tag obtained from each peptide fragmentation in MS/MS analyses were used to search for protein candidates using Mascot version 2.2 from Matrix Science [54]. Peak intensity was used to select up to 50 peaks per spot for peptide mass fingerprinting, and 50 peaks per precursor for MS/MS identification. Tryptic autolytic fragments, keratin and matrix-derived peaks were removed from the dataset used for the database search. The searches for peptide mass fingerprints and tandem MS spectra were performed in the UniProt knowledgebase, by searching in the UniProtKB/Swiss-Prot database [24], containing 519348 entries. Fixed and variable modifications were considered (Cys as *S*-carbamidomethyl derivate and Met as oxidized methionine, respectively), allowing one trypsin missed cleavage site and a mass tolerance of 50 ppm. For MS/MS identifications, a precursor tolerance of 50 ppm and MS/MS fragment tolerance of 0.3 Da were used. Identifications were accepted as positive when at least five peptides matched and at least 20% of the peptide coverage of the theoretical sequences matched within a mass accuracy of 50 or 25 ppm with internal calibration. Probability scores were significant at *p* < 0.01 for all matches.

### 3.8. Cisplatin Resistance

Sensitivity to the cytotoxic effect of cisplatin was assessed using a colony formation assay. Cultures in 1 mL SD containing a total of 6 × 10^6^ cells were exposed for 4 h to cisplatin at concentrations of 0, 0.5, 1.0, 2.0, and 2.5 and 5 mM, washed once in phosphate-buffered saline (PBS), re-suspended, diluted 1:4000, and plated onto YPD agar plates (1% yeast extract, 2% peptone, 2% dextrose, 2% agar). After 2 days of growth at 30 °C the number of colonies was counted. The IC_50_ was defined as the drug concentration that reduced the number of colony-forming units to 50% of the value in a control culture not exposed to the drug. Each experiment was repeated three times with duplicate cultures for each drug concentration. A *t* test was applied to evaluate the differences between means. Statistically significant changes and their *p* values are indicated in Figure 2.

### 3.9. Fluorescence Microscopy Assay for Monitoring Mitophagy

The *S. cerevisiae* strains W303-1A and its isogenic null mutant *∆sky1* transformed with the plasmid pAS1NB:mit-Rosella [48] were grown in SD medium to mid-exponential phase. Cells were washed three times in sterile distilled water, harvested and resuspended in fresh SD media to an OD_600_ of 1. Cells were treated with cisplatin 600 or 1200 µM as explained above. Treated and control cells were washed three times with sterile distilled water and resuspended in fresh SD media to be directly spotted onto microscope slides. Cells were then immediately imaged using a Nikon Eclipse 50i equipped with a 100× oil-immersion objective. The mit-Rosella cassette contains a mitochondrial leader sequence (from citrate synthase) to target the Rosella biosensor to the mitochondrial matrix. The biosensor consists of two fluorescent proteins, pHluorin (488 nm (Ex)/508 nm (Em)) and Ds. Red T3 (543 nm (Ex)/587 nm (Em)). pHluorin does not fluoresce at low pH, so when mitochondria are located in the vacuole (pH~5.5) it fluoresces only red. Delivery of Rosella to the vacuole was quantified by scoring for the accumulation of red fluorescence in the vacuole concomitant with checking for the absence of green fluorescence. Green fluorescence emission (500 nm) and red fluorescence emission (620 nm) images were acquired sequentially upon excitation with a mercury lamp using green fluorescent protein (GFP) and tetramethylrhodamine (TRITC) block filters respectively. Twenty microscopic field images from each slide were counted for red and green fluorescent cells and the percentage of mitophagy was determined in each condition.

### 3.10. Fluorescence Assay to Quantify Mitophagy

The *S. cerevisiae* strains W303-1A and its isogenic null mutant *∆sky1* transformed with the plasmid pAS1NB:mit-Rosella were grown and treated with cisplatin 600 and 1200 µM as explained above. After 3 h, cells were washed three times with sterile PBS and resuspended in 20 µL of PBS containing 2 µL of 4',6-diamino-2-fenilindol (DAPI). After 10 min of incubation in the dark, cells were resuspended in PBS to 120 µL of final volume and deposited in 96 well microtiter clear bottom plates from Corning (CLS3631), suitable for fluorescent/luminescent assays. The relative fluorescence for each well was monitored at excitation/emission 488/500, 555/582, and 340/488 for pHluorin, Ds. Red T3 and DAPI respectively, in a Synergy H1 Hybrid Reader, from BioTek. Fluorescence quantification was normalized using DAPI as reference. Three biological replicates and three technical replicates of each were done.

### 3.11. Flow Cytometry Apoptotic Assay

The *S. cerevisiae* strains W303-1A and its isogenic null mutant ∆*sky1* were grown in SD medium to mid-exponential phase. Cells were diluted in fresh SD media to an OD_600_ of 1 and cisplatin treatment was carried out as previously explained to a final concentration of 600 µM. The percentage of apoptosis and necrosis was evaluated by means of flow cytometry analysis of YO-PRO^®^-1 and PI double staining using the Vybrant Apoptosis Assay Kit from Invitrogen (Carlsbad, CA, USA) according to the provided protocol. Briefly, after treatments cells were washed in cold phosphate buffered saline (PBS) and suspended in PBS at 6 × 10^5^ cells/mL. One microliter of YO-PRO^®^-1 and 1 µL of PI were added to each sample and incubated for 20 min at room temperature in the dark. Flow cytometry analysis was then performed in a FACScalibur flow cytometer (Franklin Lakes, NJ, USA). At least 5 × 10^4^events were acquired, obtaining data from FL1 (YO-PRO^®^-1), and FL2 (PI) detectors. Data were analyzed using Cell Quest Pro software (Becton Dickinson, New York, NY, USA). Early apoptotic, late apoptotic and necrotic cells were expressed as the percentages of YO-PRO^®^-1+/PI− and YO-PRO^®^-1+/PI+ and YOP-RO^®^-1−/PI+ cells respectively. Measures from six biological replicates were considered for statistical analysis. Data were expressed in relative percentage of each type, attributing the 100% to the W303-1A strain without cisplatin treatment. The samples were also visualized by fluorescence microscopy.

## 4. Conclusions

Cisplatin interacts with DNA, but the inhibition of DNA replication cannot solely account for its cytotoxic activity [2]. In this study we have investigated the phosphoproteome signatures of the response of *S. cerevisiae* to the anti-carcinogenic drug cisplatin as well as the influence of the depletion of the SR kinase Sky1, also related to cisplatin resistance. Several proteins previously related to oxidative stress in yeast cells have been identified in this study. This result is in accordance with previous data showing that cisplatin induces a response to oxidative stress in other eukaryotic cells. Besides, proteins previously related to programmed cell death in yeast and mitophagy are also among proteins showing abundance changes in this study. It has been proved that deletion of one of this proteins, Bmh2, causes a significant decrease in the resistance to cisplatin. The results obtained in the proteome analysis prompted us to investigate the effect of deletion of Sky1 on programmed cell death and mitophagy. The results presented here support the participation of this protein in these cellular processes in response of cisplatin and they open the field to future studies, which contribute to elucidate the molecular mechanisms of cisplatin action and Sky1 function.

## Supplementary Files

Supplementary File 1

## References

[1] Eastman A. (1990). Alkylating and platinum-based agents. Curr. Opin. Oncol..

[2] Eastman A. (1990). Activation of programmed cell death by anticancer agents: Cisplatin as a model system. Cancer Cells.

[3] Florea A.M., Busselberg D. (2011). Metals and breast cancer: Risk factors or healing agents?. J. Toxicol..

[4] Basu A., Krishnamurthy S. (2010). Cellular responses to cisplatin-induced DNA damage. J. NucleicAcids.

[5] Burger H., Capello A., Schenk P.W., Stoter G., Brouwer J., Nooter K. (2000). A genome-wide screening in *Saccharomyces cerevisiae* for genes that confer resistance to the anticancer agent cisplatin. Biochem. Biophys. Res. Commun..

[6] Huang R.Y., Eddy M., Vujcic M., Kowalski D. (2005). Genome-wide screen identifies genes whose inactivation confer resistance to cisplatin in *Saccharomyces cerevisiae*. Cancer Res..

[7] Cunha D., Cunha R., Corte-Real M., Chaves S.R. (2013). Cisplatin-induced cell death in *Saccharomyces cerevisiae* is programmed and rescued by proteasome inhibition. DNA Repair.

[8] Schenk P.W., Boersma A.W., Brandsma J.A., den Dulk H., Burger H., Stoter G., Brouwer J., Nooter K. (2001). *SKY1* is involved in cisplatin-induced cell kill in *Saccharomyces cerevisiae*, and inactivation of its human homologue, SRPK1, induces cisplatin resistance in a human ovarian carcinoma cell line. Cancer Res..

[9] Siebel C.W., Feng L., Guthrie C., Fu X.D. (1999). Conservation in budding yeast of a kinase specific for SR splicing factors. Proc. Natl. Acad. Sci. USA.

[10] Yun C.Y., Fu X.D. (2000). Conserved SR protein kinase functions in nuclear import and its action is counteracted by arginine methylation in *Saccharomyces cerevisiae*. J. Cell Biol..

[11] Windgassen M., Krebber H. (2003). Identification of Gbp2 as a novel poly(A)+ RNA-binding protein involved in the cytoplasmic delivery of messenger RNAs in yeast. EMBO Rep..

[12] Porat Z., Erez O., Kahana C. (2006). Cellular localization and phosphorylation of Hrb1p is independent of Sky1p. Biochim. Biophys. Acta.

[13] Nolen B., Yun C.Y., Wong C.F., McCammon J.A., Fu X.D., Ghosh G. (2001). The structure of Sky1p reveals a novel mechanism for constitutive activity. Nat. Struct. Biol..

[14] Papoutsopoulou S., Nikolakaki E., Giannakouros T. (1999). SRPK1 and LBR protein kinases show identical substrate specificities. Biochem. Biophys. Res. Commun..

[15] Edmond V., Moysan E., Khochbin S., Matthias P., Brambilla C., Brambilla E., Gazzeri S., Eymin B. (2011). Acetylation and phosphorylation of SRSF2 control cell fate decision in response to cisplatin. EMBO J..

[16] Schenk P.W., Boersma A.W., Brok M., Burger H., Stoter G., Nooter K. (2002). Inactivation of the *Saccharomyces cerevisiae SKY1* gene induces a specific modification of the yeast anticancer drug sensitivity profile accompanied by a mutator phenotype. Mol. Pharmacol..

[17] Erez O., Kahana C. (2001). Screening for modulators of spermine tolerance identifies Sky1, the SR protein kinase of *Saccharomyces cerevisiae*, as a regulator of polyamine transport and ion homeostasis. Mol. Cell. Biol..

[18] Forment J., Mulet J.M., Vicente O., Serrano R. (2002). The yeast SR protein kinase Sky1p modulates salt tolerance, Membrane potential and the Trk1,2 potassium transporter. Biochim. Biophys. Acta.

[19] Deka P., Bucheli M.E., Moore C., Buratowski S., Varani G. (2008). Structure of the yeast SR protein Npl3 and interaction with mRNA 3'-end processing signals. J. Mol. Biol..

[20] Totallab. http://www.nonlinear.com/products/progenesis/samespots/.

[21] Amoutzias G.D., He Y., Lilley K.S., van de Peer Y., Oliver S.G. (2012). Evaluation and properties of the budding yeast phosphoproteome. Mol. Cell. Proteomics.

[22] Huber A., Bodenmiller B., Uotila A., Stahl M., Wanka S., Gerrits B., Aebersold R., Loewith R. (2009). Characterization of the rapamycin-sensitive phosphoproteome reveals that Sch9 is a central coordinator of protein synthesis. Genes Dev..

[23] Albuquerque C.P., Smolka M.B., Payne S.H., Bafna V., Eng J., Zhou H. (2008). A multidimensional chromatography technology for in-depth phosphoproteome analysis. Mol. Cell. Proteomics.

[24] Swiss-Prot. http://www.expasy.ch/sprot.

[25] Ghaemmaghami S., Huh W.K., Bower K., Howson R.W., Belle A., Dephoure N., O’Shea E.K., Weissman J.S. (2003). Global analysis of protein expression in yeast. Nature.

[26] ProtParam. http://web.expasy.org/protparam/.

[27] Kastanos E.K., Woldman Y.Y., Appling D.R. (1997). Role of mitochondrial and cytoplasmic serine hydroxymethyltransferase isozymes in *de novo* purine synthesis in *Saccharomyces cerevisiae*. Biochemistry.

[28] Konrad M. (1992). Cloning and expression of the essential gene for guanylate kinase from yeast. J. Biol. Chem..

[29] Takahashi H., McCaffery J.M., Irizarry R.A., Boeke J.D. (2006). Nucleocytosolic acetyl-coenzyme a synthetase is required for histone acetylation and global transcription. Mol. Cell.

[30] Pujol N., Bonet C., Vilella F., Petkova M.I., Mozo-Villarias A., de la Torre-Ruiz M.A. (2009). Two proteins from *Saccharomyces cerevisiae*: Pfy1 and Pkc1, play a dual role in activating actin polymerization and in increasing cell viability in the adaptive response to oxidative stress. FEMS Yeast Res..

[31] Kowalski D., Pendyala L., Daignan-Fornier B., Howell S.B., Huang R.Y. (2008). Dysregulation of purine nucleotide biosynthesis pathways modulates cisplatin cytotoxicity in *Saccharomyces cerevisiae*. Mol. Pharmacol..

[32] Petti A.A., Crutchfield C.A., Rabinowitz J.D., Botstein D. (2011). Survival of starving yeast is correlated with Oxidative stress response and nonrespiratory mitochondrial function. Proc. Natl. Acad. Sci. USA.

[33] Lee J., Spector D., Godon C., Labarre J., Toledano M.B. (1999). A new antioxidant with Alkyl hydroperoxide defense properties in yeast. J. Biol. Chem..

[34] Kim I.S., Sohn H.Y., Jin I. (2011). Adaptive stress response to menadione-induced Oxidative stress in *Saccharomyces cerevisiae* KNU5377. J. Microbiol..

[35] McDonagh B., Ogueta S., Lasarte G., Padilla C.A., Barcena J.A. (2009). Shotgun redox proteomics identifies specifically modified cysteines in key metabolic enzymes under oxidative stress in *Saccharomyces cerevisiae*. J. Proteomics.

[36] Santos N.A., Bezerra C.S., Martins N.M., Curti C., Bianchi M.L., Santos A.C. (2008). Hydroxyl radical scavenger ameliorates cisplatin-induced nephrotoxicity by preventing oxidative stress, redox state unbalance, impairment of energetic metabolism and apoptosis in rat kidney mitochondria. Cancer Chemother. Pharmacol..

[37] Martins N.M., Santos N.A., Curti C., Bianchi M.L., Santos A.C. (2008). Cisplatin induces mitochondrial oxidative stress with resultant energetic metabolism impairment, membrane rigidification and apoptosis in rat liver. J. Appl. Toxicol..

[38] Lucibello M., Gambacurta A., Zonfrillo M., Pierimarchi P., Serafino A., Rasi G., Rubartelli A., Garaci E. (2011). TCTP is a critical survival factor that protects cancer cells from oxidative stress-induced cell-death. Exp. Cell Res..

[39] Rinnerthaler M., Jarolim S., Heeren G., Palle E., Perju S., Klinger H., Bogengruber E., Madeo F., Braun R.J., Breitenbach-Koller L. (2006). MMI1 (YKL056c, TMA19), the yeast orthologue of the translationally controlled tumor protein (TCTP) has apoptotic functions and interacts with both microtubules and mitochondria. Biochim. Biophys. Acta.

[40] Farah M.E., Sirotkin V., Haarer B., Kakhniashvili D., Amberg D.C. (2011). Diverse protective roles of the actin cytoskeleton during oxidative stress. Cytoskeleton.

[41] Smolka M.B., Albuquerque C.P., Chen S.H., Zhou H. (2007). Proteome-wide identification of *in vivo* targets of DNA damage checkpoint kinases. Proc. Natl. Acad. Sci. USA.

[42] Walter W., Clynes D., Tang Y., Marmorstein R., Mellor J., Berger S.L. (2008). 14-3-3 interaction with histone H3 involves a dual modification pattern of phosphoacetylation. Mol. Cell. Biol..

[43] Tkach J.M., Yimit A., Lee A.Y., Riffle M., Costanzo M., Jaschob D., Hendry J.A., Ou J., Moffat J., Boone C. (2012). Dissecting DNA damage response pathways by analysing protein localization and abundance changes during DNA replication stress. Nat. Cell Biol..

[44] Burbelo P.D., Hall A. (1995). 14-3-3 proteins hot numbers in signal transduction. Curr. Biol..

[45] Clapp C., Portt L., Khoury C., Sheibani S., Norman G., Ebner P., Eid R., Vali H., Mandato C.A., Madeo F. (2012). 14-3-3 protects against stress-induced apoptosis. Cell. Death Dis..

[46] Jazwinski S.M., Kriete A. (2012). The yeast retrograde response as a model of intracellular signaling of mitochondrial dysfunction. Front. Physiol..

[47] Jazwinski S.M. (2012). The retrograde response and other pathways of interorganelle communication in yeast replicative aging. Subcell. Biochem..

[48] Rosado C.J., Mijaljica D., Hatzinisiriou I., Prescott M., Devenish R.J. (2008). Rosella: A fluorescent pH-biosensor for reporting vacuolar turnover of cytosol and organelles in yeast. Autophagy.

[49] Rodríguez Lombardero S., Vizoso Vázquez A., Rodríguez Belmonte E., González Siso M.I., Cerdán M.E. (2012). *SKY1* and *IXR1* interactions, their effects on cisplatin and spermine resistance in *Saccharomyces cerevisiae*. Can. J. Microbiol..

[50] Euroscarf. http://web.uni-frankfurt.de/fb15/mikro/euroscarf/.

[51] Zitomer R.S., Hall B.D. (1976). Yeast cytochrome C messenger RNA *in vitro* translation and specific immunoprecipitation of the *CYC1* gene product. J. Biol. Chem..

[52] Ruiz-Romero C., Lopez-Armada M.J., Blanco F.J. (2006). Mitochondrial proteomic characterization of human normal articular chondrocytes. Osteoarthr. Cartil..

[53] Sechi S., Chait B.T. (1998). Modification of cysteine residues by alkylation. A tool in peptide mapping and protein identification. Anal. Chem..

[54] Matrixscience. http://web.uni-frankfurt.de/fb15/mikro/euroscarf/.

