# Supplementary material for: Proteomic Analyses Reveal that Sky1 Modulates Apoptosis and Mitophagy in Saccharomyces cerevisiae Cells Exposed to Cisplatin
Source: Int J Mol Sci. 2014 Jul 15;15(7):12573–90. doi: 10.3390/ijms150712573 (PMC4139861; doi:10.3390/ijms150712573)
Supplement: Supplementary File 1 [file ijms-15-12573-s001.pdf]

Supplementary Information

Table S1. Changes in mRNA levels observed for genes encoding proteins included in Table 1. Data are from accession GEO (GSE41094): * + activation; − repression.

**Untreated Mutant Δ*sky1* *versus* Untreated Wild Type**			
**Gene**	**W303-1A $\log_2$ Mean ± SD**	**Δ*sky1* $\log_2$ Mean ± SD**	**FOLD ***
YLR179C	7.78 ± 0.02	7.79 ± 0.03	1.00
ACS2; YLR153C	9.24 ± 0.30	9.61 ± 0.07	1.45
SHM2; YLR058C	9.62 ± 0.11	9.16 ± 0.08	−1.58
GUK1; YDR454C	7.93 ± 0.04	8.35 ± 0.08	1.53
PFY1; YOR122C	9.44 ± 0.05	10.18 ± 0.12	2.09
**Wild Type Treated *versus* Untreated**			
**Gene**	**W303-1A $\log_2$ Mean ± SD**	**W303-1A + cisPt $\log_2$ Mean ± SD**	**FOLD ***
MET6; YER091C	10.64 ± 0.14	11.68 ± 0.03	2.83
LYS20; YDL182W	8.46 ± 0.10	7.95 ± 0.03	−1.66
ADO1; YJR105W	8.79 ± 0.10	8.35 ± 0.06	−1.55
ADH1; YOL086C	12.02 ± 0.08	11.83 ± 0.08	−1.20
ARO4; YBR249C	7.52 ± 0.14	7.08 ± 0.04	−1.55
**Mutant Δ*sky1* Treated *versus* Untreated**			
**Gene**	**Δ*sky1* $\log_2$ Mean ± SD**	**Δ*sky1* + cisPt $\log_2$ Mean ± SD**	**FOLD ***
UGP1; YKL035W	11.34 ± 0.03	11.47 ± 0.05	1.14
PRB1; YEL060C	10.55 ± 0.06	10.91 ± 0.02	1.44
STI1; YOR027W	10.33 ± 0.07	10.38 ± 0.05	1.05
BMH1; YER177W	10.76 ± 0.02	10.66 ± 0.10	−1.11
BMH2; YDR099W	10.34 ± 0.06	10.43 ± 0.09	1.09
TMA19; YKL056C	10.89 ± 0.20	10.57 ± 0.10	−1.39
YLR179C	7.79 ± 0.03	7.62 ± 0.06	−1.19
GUK1; YDR454C	8.35 ± 0.08	8.19 ± 0.04	−1.18
ADO1; YJR105W	8.85 ± 0.03	8.40 ± 0.08	−1.57
**Treated Mutant Δ*sky1* *versus* Treated Wild Type**			
**Gene**	**W303-1A + cisPt $\log_2$ Mean ± SD**	**Δ*sky1* + cisPt $\log_2$ Mean ± SD**	**FOLD ***
HSP26; YBR072W	10.86 ± 0.10	11.15 ± 0.05	1.33
AHP1; YLR109W	11.81 ± 0.04	11.68 ± 0.08	−1.15
YLR179C	7.71 ± 0.05	7.62 ± 0.06	−1.10

Table S2. Cellular abundance and stability of the identified proteins and Sky1 known substrates.

Spot [&]	ID [$]	Gene	Molecules/Cell	Instability Index (*)	Classification
290	2	*ACS2; YLR153C*	225,000	32.03	stable
310	3	*MET6; YER091C*	264,000	33.76	stable
341	6	*STI1; YOR027W*	67,600	39.66	stable
475	8	*UGP1; YKL035W*	17,200	31.06	stable
521	9	*SHM2; YLR058C*	67,600	27.27	stable
610	10a	*ADH1; YOL086C*	not reported	20.71	stable
610	10b	*ARO4; YBR249C*	26,300	34.92	stable
537	11	*LYS20; YDL182W*	28,100	32.87	stable
684	15	*ADO1; YJR105W*	22,200	21.59	stable
924	19	*YLR179C*	6230	34.17	stable
1052	20	*AHP1; YLR109W*	16,200	34.33	stable
824	21	*PRB1; YEL060C*	1600	32.36	stable
1010	23	*GUK1; YDR454C*	20,500	21.06	stable
865	32	*HSP26; YBR072W*	19,300	48.11	unstable
1293	36	*PFY1; YOR122C*	not reported	35.55	stable
761	41	*BMH1; YER177W*	158,000	57.99	unstable
927	42	*TMA19; YKL056C*	27,800	22.18	stable
Sky1 substrate		*NPL3;YDR432W*	not reported	66.14	unstable
Sky1 substrate		*GBP2;YCL011C*	2540	44.70	unstable

[&] Master protein spot number acording to black numbers in Figure 1; [$] Identification protein number according to red number in Figure 1; * In log phase SD medium.

Figure S1. Correlation of predicted and experimental pI.

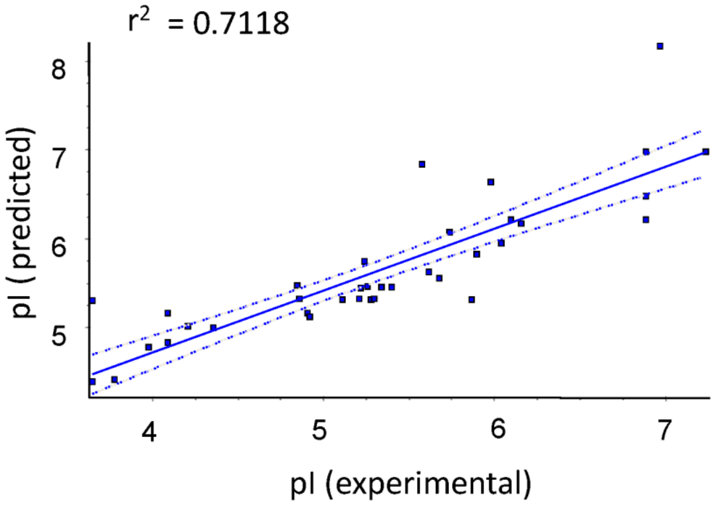

Figure S2. Control analyses and DIGE design. (**A**) Mono-dimensional silver-stained gel showing the different samples. 1–3, W303-1A without treatment; 4–6, W303-1A treated with cisplatin; 7–9, Δ*sky1* without treatment; 10–12, Δ*sky1* treated with cisplatin; (**B**) standard silver-stained DIGE gel; (**C**) Design of dyes staining.

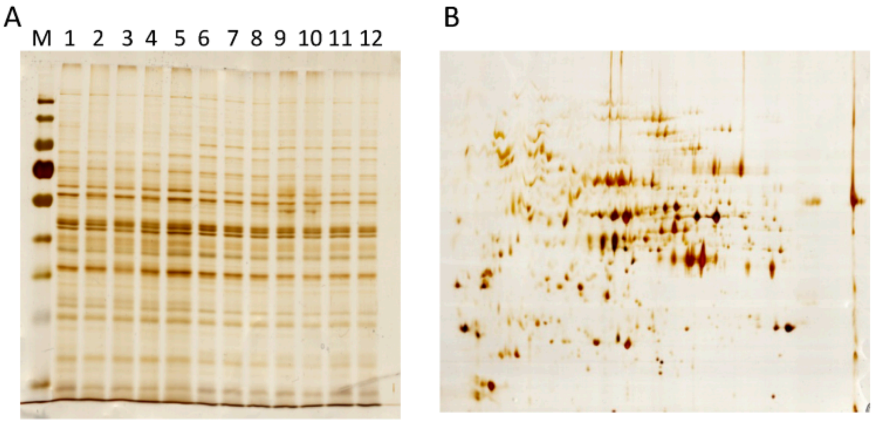

C

DIGE sample distribution	Cy2	Cy3	Cy5
Gel1	Pool IS	Wt-NT1	Mut-NT1
Gel2	Pool IS	Mut-T1	Wt-NT2
Gel3	Pool IS	Wt-NT3	Wt-T1
Gel4	Pool IS	Mut-NT2	Mut-T2
Gel5	Pool IS	Wt-T2	Mut-NT3
Gel6	Pool IS	Mut-T3	Wt-T3
Wt, W303; Mut, Δ*sky1*; T cisplatin added; NT without cisplatin			
